# Organizing workplace health literacy to reduce musculoskeletal pain and consequences

**DOI:** 10.1186/s12912-015-0096-4

**Published:** 2015-09-17

**Authors:** Anne Konring Larsen, Andreas Holtermann, Ole Steen Mortensen, Laura Punnett, Morten Hulvej Rod, Marie Birk Jørgensen

**Affiliations:** National Research Centre for the Working Environment, Lersø Parkallé 105, 2100 Copenhagen Ø, Denmark; Department of Occupational Medicine, Copenhagen University Hospital Holbæk, 4300 Holbæk, Denmark; University of Massachusetts Lowell, One University Avenue, Lowell, MA 01854 USA; National Institute of Public Health, University of Southern Denmark, Øster Farimagsgade 5A, 1353 Copenhagen K, Denmark

## Abstract

**Background:**

Despite numerous initiatives to improve the working environment for nursing aides, musculoskeletal disorders (pain) is still a considerable problem because of the prevalence, and pervasive consequences on the individual, the workplace and the society. Discrepancies between effort and effect of workplace health initiatives might be due to the fact that pain and the consequences of pain are affected by various individual, interpersonal and organizational factors in a complex interaction. Recent health literacy models pursue an integrated approach to understanding health behavior and have been suggested as a suitable framework for addressing individual, organizational and interpersonal factors concomitantly. Therefore, the aim of the trial is to examine the effectiveness of an intervention to improve health literacy (building knowledge, competences and structures for communication and action) at both the organizational and individual level and reduce pain among nursing aides.

**Methods/design:**

The intervention consists of 2 steps: 1) Courses at the workplace for employees and management in order to organize a joint fundament of knowledge and understanding, and a platform for communication and action about pain prevention in the organization. 2) Organizing a fixed 3-weekly structured dialogue between each employee and her/his supervisor, with particular focus on developing specific plans to prevent and reduce pain and its consequences. This enables the workplace to generate knowledge about employee resources and health challenges and to act and convey this knowledge into initiatives at the workplace.

**Discussion:**

Previous studies to improve health literacy have primarily targeted patients or specific deprived groups in health care or community settings. Recently the idea of the workplace as an arena for improving health literacy has developed emphasizing the organizational responsibility in facilitating and supporting that employees obtain basic knowledge and information needed to understand and take action on individual and occupational health concerns. The literature about workplace health literacy is very limited but points at the importance of educating employees to be able to access, appraise and apply health information and of organizing the infrastructure and communication in the organization. This study suggests a concrete operationalization of health literacy in a workplace setting. Results are expected published in 2016.

## Background

Musculoskeletal disorders (pain) are a considerable societal problem because of the prevalence, the pervasive impact on the individual, and the scope of the individual and socio-economic consequences. The consequences include reduced quality of life for the individual, increased sickness absence at the workplace, and economic consequences for the society [1–3]. Within short educated job groups there is a high prevalence of pain, especially within job groups with high physically demanding work [4]. For example, the annual prevalence of low back pain among nursing aides is reported to be more than 60 % in Denmark [5]. Pain may lead to functional impairments, and the combination with physically demanding work tasks, such as patient handling in health care institutions and home care, substantially increase the risk of consequences of pain such as impaired quality of life, work disability, sickness absence and early exit from the labor market [2, 3, 6–8].

During the past decade, management in Danish nursing homes and home care has initiated a number of initiatives to improve the working environment, such as “no lift programs” or programs to increase employee involvement in decision-making and in health and safety issues [9]. Despite these efforts, the workers still report heavy lifting, high physical and emotional work demands, and an inadequate decision-latitude (high degrees of responsibility concomitant with low decision-latitude) [10, 11]. Thus, there is not a clear connection between the work environment efforts reported from management and experiences of employees. Discrepancies between effort and effect of workplace health initiatives is a well-known issue, with implementation challenges reported by several in the past decade [12, 13]. Problems with compliance and participation are recognized challenges in studies with low-educated workers in workplace settings [14–17], increasing the risk of low effectiveness [18, 19] in groups with the most severe occupational exposures and health needs.

Implementation challenges could be due to the design of the interventions aiming to target only one level of the organization (the employees) and attempting to isolate a single risk factor (for example, to reduce heavy lifting, reduce highly repetitive work, or improve physical fitness), thereby not taking into account the complex interaction of individual and organizational factors that can affect health behavior as well as the incidence, recurrence and persistence of pain. For low-income workers, relevant individual and contextual factors may include limited formal education and lack of knowledge about fundamental health issues and limited competences to understand, interpret and use health information i.e., low individual health literacy [20, 21].

**Fig. 1:**
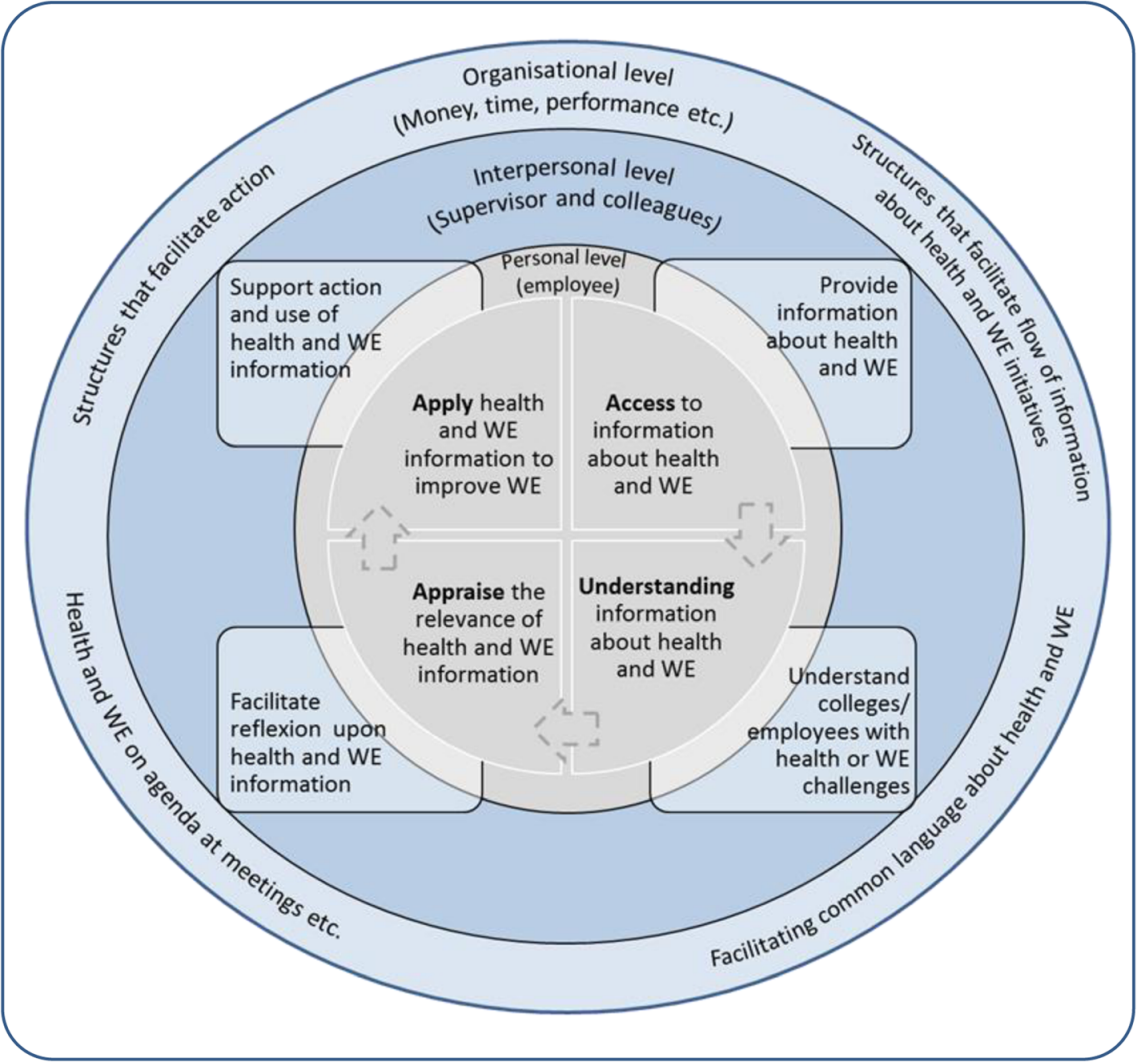
The conceptual model of the study. The individual employee’s ability to access, understand, appraise and apply information about health and work environment is affected by personal, interpersonal and organizational determinants. WE: Work Environment

In a workplace setting, health literacy has been associated with individual health behaviors such as knowledge and management of pain and non-medication modes of treating pain [22, 23] and the confidence about the ability to influence health or working conditions [24]. At the organizational level, working conditions (e.g. employee influence, work pace and work tasks) can affect the workers’ possibilities to apply their health behavior competences in the daily work routines. Therefore, to address these individual and organizational factors concomitantly and also their interconnectedness, it is necessary to pursue an integrated intervention approach with multiple facets (building knowledge, competences and structures for communication and action) at both the organizational and the individual level (Fig. 1) [25–28].

### Health literacy in a workplace setting

Recent health literacy models pursue such an integrated approach to understanding health behavior and have been suggested as a suitable framework for addressing individual, organizational and interpersonal factors [21]. Health literacy is based on theories concerning causes for inequity in health and builds on the assumption that different environments create different premises for maintaining health [29]. Health literacy incorporates the individuals’ opportunities for prevention (defined as the individual’s opportunities and capabilities to access, understand, process and use health information), and the potential influence of environmental and interpersonal factors on these capabilities [21]. Examples of interventions aimed at the individual level include self-management programs and education programs such as cognitive behavioral training [30–32].

Recently, the importance of the workplace as an arena for facilitating employee health literacy has been emphasized [33]. Workplace health literacy entails an organization where 1) employees and managers have a common level of knowledge about prevention and handling of work environment challenges and pain, 2) structures for communicating about work environment and health across all levels in the organization, are provided and 3) structures and management facilitate and enable relevant action [23, 29, 33]. Therefore, workplace health literacy may be a suitable theoretical framework for interventions for low-income workers, empowering both the individual employees and the management with knowledge and competences to prevent and handle pain and consequences of pain effectively and furthermore building organizational structures that enable communication about work environment and pain, and facilitate action.

In this study we aim to operationalize workplace health literacy by 1) providing managers and supervisors with knowledge about prevention and handling of employees with pain, tools to communicate with employees about work environment challenges and tools to act; 2) providing employees with knowledge about prevention and handling of pain and competences to communicate and act upon work environment challenges in relation to pain; and 3) organizing work processes and structures (such as work organization, ergonomics and meetings) that enable a continuous information flow between employees and supervisors, with the purpose of identifying and initiating relevant actions for improving employee work environment and health.

Interventions on knowledge, work organization and communication are complex and require a close understanding of the workplace culture and normal routines, structures, and communicative processes [34]. Before development and implementation of a complex intervention, a comprehensive needs assessment is necessary [35, 36], to optimize the tailoring of the intervention content and implementation to the specific needs and resources at each workplace [34, 37, 38].

The main aim of this paper is to describe the rationale, contents and design of an organizational intervention trial to strengthen workplace health literacy in nursing homes. The aim of the trial is to examine the effectiveness of an intervention consisting of 2 steps: 1) Courses at the workplace for employees and management in order to organize a joint fundament of knowledge and understanding, and a platform for communication and action about pain prevention in the organization. 2) Organizing a fixed 3-weekly structured dialogue between each employee and her/his supervisor, with particular focus on how each can contribute to developing specific plans to prevent and reduce pain and its consequences.

The study has three main hypotheses. These are that the intervention will: 1) increase workplace health literacy (knowledge, competences, communication and structures for action) (secondary outcome); 2) reduce employee pain (primary outcome); 3) reduce consequences of pain (sickness absence, pain-related sickness absence, bothersomeness and fear avoidance) (secondary outcomes).

## Methods

### Recruitment of workplaces

The majority of the Danish nursing homes are owned by the municipalities. We aim to recruit one municipality where the majority of the nursing homes agree to participate in the intervention, making it possible to include the whole organization (employees, supervisors and managers at the nursing homes but also representatives from the municipality including the responsible director of care in the municipality) in the intervention.

### Participants/study population

The participants in the study will be employees in elderly care in nursing homes in one municipality. The main employees in the elderly care in the municipality are nurses’ aides who are either trained social - and health service (SHS) aides or helpers. The managers are primarily nurses. In Denmark, SHS helpers have 14 months of training, SHS aides have an additional 6 months of training. All employees working in the nursing homes are eligible to participate in the study. It is a workplace decision to participate in the intervention which is conducted fully during work hours. All permanent staff at each workplace - including the kitchen, cleaning and technical staff – will be part of the intervention. Thus, the study population consists of short-educated service- and blue-collar workers in elderly care, but will be referred to as nurses’ aides because the majority of the employees belong to this profession. Whether temporary staffs are such an embedded part of the organization that they should participate in the intervention will be decided by the manager at each workplace. Supervisors and top management will also participate in the intervention.

### The intervention

The objective is to develop workplace health literacy in the nursing homes to reduce pain and consequences of pain among employees. The aim is to generate workplace knowledge about the balance between employee resources and health challenges, facilitate clear communication about employee health and work situation, and enable the workplace to act and convey this knowledge into possibilities and initiatives at the workplace.

**Fig. 2:**
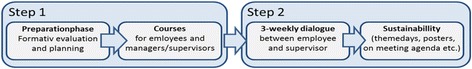
The two steps of the intervention and the components within each step

The intervention consists of two steps (1. Preparation and knowledge building; 2. Structured communication and maintenance), each containing two components (see Fig. 2). The first step, Preparation and knowledge building, contains a) a preparation phase that includes a four-stepped formal evaluation of the existing framework for handling employees with pain and tailoring of the intervention to each specific workplace b) delivery of courses. The courses provide a joint fundament of knowledge about prevention and handling of pain in the workplace setting and tools for improved communication for the managers and employees. The courses consist of 2 initial courses, followed by “booster”courses every half year for 2 years.

The second step, Structured communication and maintenance, introduces structured communication between the supervisor and the employee every 3rd week for 12 months to secure transfer of the acquired knowledge to preventive and pain reducing behavior. Additional initiatives will be taken continuously throughout the intervention period to secure program sustainability at all levels in the organization. The steps and content are described more thoroughly below.

#### The content

### Step 1: Preparation phase and knowledge building

#### A preparation phase with focus on how to integrate and maintain workplace health literacy in the normal procedures at the workplace to reduce pain and consequences of pain

Within the first step, the first component is the preparation phase. This phase consists of the tailoring of the intervention to the nurses’ aides. For this purpose a thorough needs assessment will be made, using existing registrations of the working environment at the nursing homes and relevant scientific literature. To further tailor the intervention to the specific workplaces, the activities will be specified and adjusted using Normalization Process Theory (NPT) [34]. In this process, representatives from all levels of the organization will be involved in the development, adjustment and implementation of the intervention. Within this component the following four steps will be taken.

### Evaluation of the workplace using NPT

A thorough formative evaluation of the workplaces to evaluate the workplace readiness for this intervention and to optimize the tailoring of the intervention to the specific needs and resources at each workplace [34, 37, 38], will be made before the start of the intervention using NPT. NPT is a framework for developing, evaluating and implementing complex interventions [34]. The use of NPT will be described in more detail below, in the “process evaluation” section.

All levels of the organization will be involved in the formative evaluation at each workplace. First, a meeting will be held at each workplace with the manager, representatives for the team leaders, and employees including health and safety representatives and union representatives. The planning of the study will be discussed and specific issues for each workplace will be considered in the specific tailoring of the intervention into the workplace.

This will be followed by direct communication with the employees. The researchers will provide information about the project first through e-mails and then orally and through posters and brochures. They will hold open meetings where employees have the opportunity to ask questions about the intervention and evaluation of the study.

Representatives of all levels of the organization will be interviewed 1-2 months before the start of the courses, in the form of qualitative interviews with managers and focus groups with all supervisors plus selected employee representatives. The interviews will uncover the existing framework for supporting employees with health and work environment challenges (e.g. the workplace procedures for employees with chronic diseases or pain, possibilities to adjust work routines, health promotion initiatives, and possibilities for employees regarding health care specialists such as physiotherapist, psychologists) as well as possible barriers and expectations. Furthermore the interviews will help clarify the role of the manager, supervisors and employees in the intervention and in each of the steps of the intervention.

On the basis of these data, a resource assessment and a business case for each workplace will be developed in cooperation between the researchers and workplace representatives and presented to all employees at the workplace. The resource assessment will identify the existing support system in the workplace. The business case identifies barriers and possibilities for successful implementation and captures the local workplace objectives for engaging in the intervention.

### Obtaining organizational commitment

Organizational commitment will be obtained through broad information from the researchers about the elements of the intervention and from the workplace (employees, supervisors and management) to the researcher about the workplace and their needs and resources. The director of Health and Care, managers of the nursing homes, the work environment representatives, union representatives, supervisors and the workers are all expected to participate active in the adjusting and implementation of the intervention. The reason (s) of each group for participating in the intervention will be identified and representatives from each group will be involved in developing a business case that expresses the common objectives for participating. The business case will be communicated to all employees by posters and meetings. The business case expresses the link between the intervention and the local objectives of both employee and management. The aim is that the employees feel ownership by recognizing their own words and objectives on the business case posters.

### Forming a steering group and local working groups

A steering committee will be formed in each participating municipality. The steering committee will consist of representatives from the municipality, representatives from all participating workplaces, and local union representatives. Furthermore local workgroups will be formed - primarily based on already existing working groups - consisting of local work environment representatives, local union representatives and management representatives. These groups will deal with issues at the overall or local level respectively.

### Local coordinators at the workplace

At each workplace the workgroup will select one or two coordinators among the employees. Their main tasks will be liaising with the researchers and the steering group and also handling, initiating and motivating the on-going initiatives at the workplace. Another important focus is identifying barriers to trust and constructive communication about health at the workplace and (if necessary) working with the researchers and/or the local work group to identify possibilities for overcoming these barriers.

#### Courses for managers and employees (2 × 3 h)

The second component in the first step consists of courses for managers and employees. The purpose of the courses is to organize a joint fundament of knowledge in the workplace to secure that employees have the knowledge and competences to access, understand, process and assess information from the organization about the work environment, pain and consequences. An additional purpose is to ensure that management provides information to employees about possibilities for handling work environment and health challenges at the workplace and supports employees in preventing and handling pain. Through knowledge about tools for constructive communication, the aim is to improve workplace communication and the flow of information about work environment, prevention and handling of pain and consequences so that relevant actions can be taken.

Two initial courses of 3 h and a 3 h “booster”-course will be held every half year for both the employees and managers/supervisors. The two initial courses are based on Cognitive Behavioral Therapy (CBT), using a modified version of the program developed by Linton [31], further developed for a working population by Jørgensen et al. [39] and previously used in an intervention among nursing aides [40]. The courses for the management and employees are carried out separately due to small differences in the content of the course material and more importantly to create the best conditions for constructive discussions among employees and supervisors separately about a topic that can be difficult to talk about openly. However, the courses follow the same structure with a short lecture on the themes, problem-solving training and training of new skills (e.g. analyzing dilemmas and identifying solutions).

#### General content of the courses

The first course will focus on improving the participants’ understanding of pain, increase the awareness of the experience of pain and the anticipation of pain by performing cognitive exercises on how physical activity may negatively or positively relate to pain. Another main focus will be on pain in relation to physically demanding work. The second course will focus on strategies for coping, tools for improving communication, and the ability to function and have a good life quality despite pain. Moreover, the positive long-term effects from appropriate pain coping will be discussed among the participants [31]. Each employee receives a workbook containing a description of the main concepts and tools that are taught in the courses. These workbooks have the purpose of making it possible for the participants to refresh the new skills and facilitate the use of these skills in their everyday life and in the dialogue with their manager.

Experienced instructors are trained to carry out the intervention activities. The intervention will be delivered at each workplace, and the delivery of the courses will be guided by a written intervention protocol describing the content, structure and aim of each of the four different courses (employee and management first and second course).

#### Specific content of management courses

All supervisors and the manager at each workplace participate in the management courses. The primary focus in these courses will be on how to understand, recognize and cope with employees with pain, and tools for communication and action. The managers will be trained in how to implement a trust-based and appreciative dialogue with each employee about health and work environment. Furthermore the existing framework for prevention and handling employees with pain will be discussed along with the possibility of including new initiatives if desired.

#### Specific content of employee courses

The courses will be carried out in groups of 10-15 employees. The local coordinators will be responsible for dividing employees into groups. The primary elements in the courses for the employees are prevention of muscle pain and strategies for coping with pain at work. Also tools for improving communication with colleagues and management about health and work will be introduced and practiced (for example, how to analyze a specific workplace situation or when to talk to a supervisor, a colleague or a personal friend). Other tools will help the employees to identify possible and relevant solutions to specific situations, analyze the consequences of different solutions, prioritize solutions and take relevant action. The courses will establish room for collegial feedback and dialogue and also build knowledge to create a platform for coordinated flow of information between employees and managers and among colleagues.

### Step 2: Integration of a new working environment tool – the dialogue

The first component in the second step is the implementation of a structured dialogue between the supervisor and each employee at 3-week intervals about work environment challenges and health. The possible topics of the dialogues are broad to ensure that a relevant dialogue can be held for all employees, with or without pain. On the basis of the joint knowledge in the organization obtained through the courses and the resource assessment in the formative evaluation, the objective of the dialogue is to optimize prevention and handling of health challenges related to work. The dialogue has three primary aims: 1) to generate a space where the employees feel comfortable to discuss work and health-related challenges, 2) to provide the supervisor with tools for facilitating a constructive dialogue focused on identifying possible solutions; and 3) Identify current work or health challenges for each employee and use knowledge from the courses to generate a plan for specific, realistic and effective actions. The plans can involve everything from participatory ergonomics (e.g. lifting equipment or changes in the organization of the work) to health-promoting initiatives such as physical or cognitive training or a combination of these.

The supervisors are responsible for scheduling the dialogue approximately every 3rd week. A tablet-based dialogue-guide is developed and all supervisors and management receive a tablet-pc for this activity. A log-on system ensures that only information about the specific employee is available on the tablet during the dialogue. The dialogue guide is tablet-based to encourage moving the dialogue out of the manager’s office.

The first time an employee is logged in, the employee is asked to answer 2 questions; 1) How much has pain affected your work during the last three weeks? (on a scale from 0 to 10) and 2) Which elements in your work do you find most challenging? (open-ended). If the employee identifies challenges at work, the employee and supervisor together develop a plan for overcoming the challenges and preventing or reducing pain and consequences of pain. The plan is registered in the dialogue guide at the tablet. The following meetings will begin with a question about to what degree the plan has been fulfilled (on a scale from 0 to 10).

The registrations are uploaded to a web-interface, available to the researchers. If the dialogue is not held the omission will be registered, making it possible for the researchers to contact the local coordinators at the specific workplace and determine how the implementation of the dialogue can be further supported prospectively.

### Sustainability initiatives to support the process

Throughout the intervention period (1 to 2 years, depending on when the nursing home steps into the intervention) initiatives will be made to motivate and engage participants. First, the concepts of the project are participatory to ensure that the intervention is tailored to the specific needs of the participants (expecting that needs change over time), facilitating ownership and motivation to sustain the initiatives. Regular meetings of the entire organization will ensure that all are informed about the main features, purposes and processes of the project. The researchers will encourage health and work environment issues to be on the agenda in multiple settings (e.g. at staff meetings, in the team coordination) and continually facilitate initiatives to remind the supervisors and employees about the key messages. This will for example be done through special events at the workplace and organizing theme weeks using posters and roll-ups at the workplace. Furthermore there is a booster session, a course of 3 h approximately every half year for both management and employees. These courses both have the purpose of following-up on the topics from the last course, to handle challenges currently at the workplace, and to prepare new employees to engage in the dialogue.

The intervention is delivered by research staff but the workplaces will be encouraged to continue both the courses and the dialogue after the intervention period.

## Study design

The study is described in accordance to the guidelines of the Trend Statement [41]. In clinical intervention research, the randomized controlled trial is considered the gold standard. However, in workplace settings this can be practically and logistically impossible and can hamper implementation due to logistical issues and impaired organizational commitment [42, 43]. Moreover, it is not possible to implement the intervention in several clusters simultaneously within the resources of the study, because of the amount of practical and logistical resources needed to implement the intervention in each workplace.

**Fig. 3:**
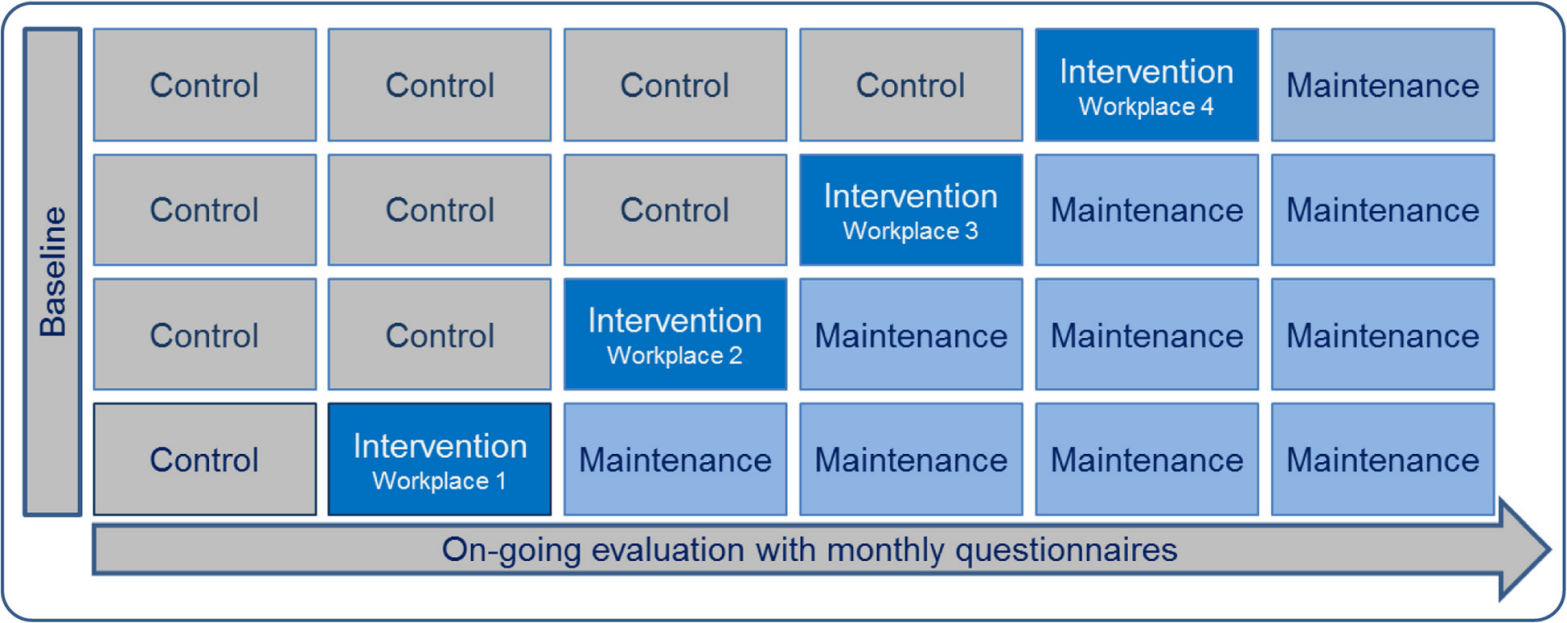
The quasi-experimental stepped-wedge intervention design, allows for repeated measures at each workplace before and after the intervention-period

These difficulties can be handled by using the more feasible, quasi-experimental stepped wedge design [42, 43], with gradual implementation of the intervention in the workplaces. A stepped-wedge design is a type of one-way crossover study in which clusters cross over from the control arm to the intervention arm at different time points (Fig. 3). Enrollment in the study will be determined between the management and the researchers. This process of enrollment will permit the researchers to focus study resources on the initial phase at one workplace at a time, to ensure that each workplace is prepared and motivated to start.

Repeated measurements will be conducted in each workplace throughout the intervention period. Baseline measures will start at the same time for all clusters, making it possible to have repetitive baseline measurements for the clusters not enrolling in the study initially.

The project has been approved by the Danish Protection Agency and is reported to the ethical committee. The local ethics committee has evaluated a description of the study and concluded that, according to Danish law as defined in Committee Act § 2, 1, an organizational intervention evaluated merely by questionnaires should not be further reported to the local ethics committee (Protocol H-1-2013 FSP). Following the committee § 1 paragraph 5 declaration of the project is therefore not relevant and the project can be implemented without further approval from the ethics committees of the Capital Region. The collection of questionnaire follows the requirements of the Helsinki declaration.

### Effect and process evaluation

**Fig. 4:**
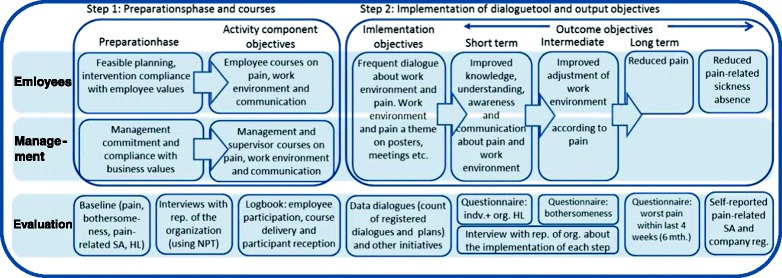
The program logic model of the study illustrates the 2 steps of the intervention and the components within these steps for both employees and management and the concurrent steps of the evaluation

A program logic model is made to illustrate all steps in the intervention and their evaluation (Fig. 4). The effect of the intervention will be measured on the primary outcome of Pain intensity (unspecified MSD) and the secondary outcomes of Bothersomeness, sickness absence, individual health literacy, organizational health literacy and fear avoidance.

#### Data collection

The data collection consists of both quantitative and qualitative measures, obtained from questionnaires collected though text messages, logbooks from instructors in the intervention, data from dialogue guide and qualitative interviews and logbooks about maintenance initiatives at each workplace.

Quantitative data will be collected through text messages delivered by the SMS Track® system [44]. The setup of the software is designed for the study in close cooperation with researchers. Every 4 weeks (on a Monday) the respondents receive an automated text message to their private mobile phone, which they are expected to answer by text message. Baseline measurements as well as repetitive measures before and after the intervention are made at all workplaces. The respondents will receive 4 questions every 4 weeks on pain intensity, bothersomeness, pain-related sickness absence and one item on organizational health literacy (providing information about possibilities for prevention and handling of pain). Every 12 weeks the respondents will receive additional 9 questions on individual and organizational health literacy and fear avoidance [45].

Before baseline, all employees will be thoroughly informed that the data collection is about to start, that it is a scientific data collection and that it is anonymous and voluntary and that they can withdraw from the data collection at any time, with no questions asked. At baseline, text message questionnaires are sent to all employees. Employees not responding to the text message will be reminded after two days with a text message reminder and if there is still no response the employee will be called by telephone to check if the phone number is correct and, if it is, whether the employee has chosen not to participate in the scientific evaluation.

Qualitative data will be collected through interviews with the municipality director of health and care, the managers at the nursing homes, focus groups with all supervisors at the workplaces and representatives from employees and employee union and work environment representatives (6-8 pers.). The interviews will be conducted before baseline and after six months. Semi-structured interview guides will be used to secure that the interviews will uncover the existing framework for supporting employees with health and work environment challenges and possible barriers and facilitators for implementation. The interview guide will make room for the respondents to contribute with thoughts relevant for the implementation and normalization of the intervention [46]. These qualitative data will be analyzed using NVivo and based on the four elements in Normalization Process Theory (NPT) (see below).

To register the participation, engagement and delivery of the courses, all instructors will complete a logbook after each course. Data from the dialogues will be available to the researchers through a web interface where all questionnaires from completed dialogues are accessible. Other logbook entries will describe all maintenance initiatives at each workplace.

#### Pain, bothersomeness and sickness absence

Because pain is often a fluctuating condition, which can be difficult to recall [47], monthly monitoring of pain, bothersomeness and pain-related sickness absence will be conducted. Pain is measured as highest intensity of pain in the muscle and joints every 4^th^ week by the text messages [48]. The respondents will be asked to answer on a 10-point scale with 0 being no pain and 10 being worst imaginable pain. The question posed is “During the previous four weeks, on a scale from 0 – 10, what was the highest intensity of pain in your muscles and joints? (0 = no pain, 10 = worst imaginable pain) [49] Pain-related sickness absence will be measured by posing the question: How many days within the past 4 weeks, have you been absent from work because of pain in muscles and joints? (Answer with a number from 0 to 28) [50].

Bothersomeness will be measured by posing the question: “How many days during the previous 4 weeks have pain in muscle and joints affected your work routines? (Answer with a number from 0 to 28) [49] In addition company-registered sick-leave will be abstracted from the management information system database.

#### Individual and organizational health literacy

Individual health literacy is operationalized as knowledge about prevention and handling pain, competences to understand, interpret and use information to improve own health and the work environment. This will be evaluated using a questionnaire validated for the specific purpose of this intervention, with items inspired by among others the Health Literacy Questionnaire (HLQ) [51], which has recently been translated into Danish. A validation of our specific questionnaire was conducted using psychometric analysis, test-retest measurements using text messages and cognitive interviews with nurses’ aides. One workplace health literacy item will be monitored monthly and the remaining items will be monitored every 12 weeks.

Organizational health literacy is defined as providing/communicating understandable information, facilitating prevention and supporting handling of health challenges. To capture health literacy at the organizational level, each employee will evaluate their supervisors’ role as a provider of understandable information, facilitator of pain prevention and supporter of handling and acting upon health challenges. Additional qualitative measures will be obtained through the process evaluation (below).

For assessment of organizational health literacy, one question covers the degree to which the supervisor helps the employee to identify options to prevent and manage pain in the workplace: How much do you agree in the following: Your supervisor helps you to identify which possibilities you have to prevent and manage pain? To capture the supervisors’ function as a health information provider, two questions will be posed: 1) How much do you agree in the following statement: Your supervisor does something active when you inform him/her about your pain? and 2) How much do you agree in the following statement: It is easy to find solutions at the workplace, if you experience pain in your body?. Three questions assess the quality of the communication between employee and supervisor: 1) How much do you agree in the following statement: When I feel pain and discomfort in the body, my supervisor really understands what I’m going through? and 2) How much do you agree in the following statement: It is easy to have good discussions about pain and discomfort with your supervisor? and 3) How much do you agree in the following statement: It is easy to get to talk to your supervisor?.

To capture the individual health literacy the following questions are posed: How much do you agree in the following statement: There are things I do regularly to prevent pain and discomfort in the body? and How much do you agree in the following statement: I am sure I have all the information I need to handle pain and discomfort in the body, the best possible way ?.

#### Fear avoidance

The effect on fear avoidance will be evaluated by one item [45]: Here is a statement about pain that others with pain problems have told us about. To what extent do you agree with the statement: I should not perform my normal activities or my normal work routines with my current level of pain (Answer with a number between 0 (completely disagree) to 10 (strongly agree)). Based on the validation of the questionnaire we decided to include this question knowing that other factors than fear avoidance can affect the answer to this question.

#### Process evaluation

The process evaluation will identify the level of implementation and the barriers and facilitators for complete implementation (i.e. normalization). Normalization process theory will be used to evaluate the process and each step in the intervention. Normalization process theory (NPT) is a framework for developing, evaluating and implementing complex interventions [34]. Through the use of NPT it is possible to identify factors that promote or inhibit the routine incorporation of complex interventions into everyday practice, i.e. normalization [52]. In addition a participatory component is recommended in needs assessments, to evaluate the workplace readiness for change and to optimize the tailoring of the intervention to the specific needs and resources at each workplace [34, 37, 38]. NPT focuses on the work that individuals and groups do to enable an intervention to become normalized.

There are four main components to NPT: coherence (or sense-making); cognitive participation (or engagement); collective action (work done to enable the intervention to happen); and reflexive monitoring (formal and informal appraisal of the benefits and costs of the intervention) [34]. These components are dynamically interconnected and interact with the wider context of the intervention, such as organizational context, structures, social norms, group processes and conventions.

In this study NPT will be used to evaluate the workplace readiness for organizational health literacy (i.e. does the intervention make sense to the employees, are they motivated to participate, how does the intervention fit into the existing structures in the organization). Likewise the tailoring of the intervention to the specific needs and resources at each workplace will be based on the workplace evaluation using NPT. Furthermore we will use the NPT in the evaluation of the process of implementing each of the elements in the intervention, to better understand the evaluation of the effect of the intervention. In the preparation phase, we will uncover the workplace readiness for organizational health literacy through interviews with employees, supervisors, management and the director of health and care in the municipality. The interviews will uncover the existing framework at the workplace for supporting employees with health and work environment challenges. Furthermore the local barriers for implementing organizational health literacy will be identified as well as existing organizational structures which can support the implementation (e.g. health promotion initiatives at the workplace, workplace meetings, organization of working environment representatives).

Approximately 6 months after the initiation of the courses, we will evaluate the process of implementing the intervention qualitatively through interviews with all levels of the organization. Here the challenges and positive factors in each of the four phases; the preparation phase, the courses, the dialogue and the maintenance phase will be uncovered retrospectively. Here the organizational health literacy will also be evaluated qualitatively. Furthermore, the employees, supervisors and managers experiences with pain, bothersomeness and sickness-absence will be uncovered.

### Courses

During the courses we will measure how many employees participate in each course. The instructors will register the execution of each course in a logbook including whether or not content was delivered according to the intervention protocol, degree of participants’ engagement, etc.

### The dialogue

We will measure the registration of completed dialogues through the web interface where the questionnaires are accessible. Furthermore we will measure the number of plans registered by employees and supervisors and to what degree the plans have been executed. The implementation of the dialogue between the employee and the manager will be evaluated qualitatively using focus group interviews with representatives from the employees and interviews with supervisors and managers. The level of concordance between the action generated by the employees and the supervisors will be compared [53].

### Maintenance initiatives

During the intervention period we will keep a log book on the initiatives at the workplace related to building or supporting organizational health literacy, such as meetings with specific health-related topics on the agenda, posters, roll ups, and health-related “theme days.”

### Sample size calculation

For sample size calculation we used the method described by Hussey et al. 2006, for the stepped wedge design [54]. The sample size is calculated for pain intensity (numeric rating scale 0-10). Pain intensity variance was set to be 2.1 based on a study by Kovacs et al. on patients with non-specific low back pain. We estimated a potential effect size of 0.5 after 6 months’ intervention. With an α of 0.05, a power of 0.8, and an intracluster correlation coefficient of 0.05, we calculated that we needed 130 participants in a stepped-wedge trial to allow analyses of effect on pain intensity. Due to a general level of 20 % in employee turnover and risk of drop-out from questionnaire survey from other reasons, we added another 20 % to the sample size. Thus a minimum of 150 individuals have to be included in the stepped-wedge-designed evaluation.

### Statistical analysis

Baseline characteristics will be described using questionnaires and register data. Analyses regarding the effectiveness of the primary outcomes and secondary outcomes will be performed after six months of intervention by means of multilevel analyses (linear mixed model (LMM) or generalized linear mixed models (GLMM)) [55]. Multilevel analyses will be used in order to take clustering of observations of workers within the same team into account, as well as repeated measurements within one participant [56].

### Handling of missing data and loss to follow up

Efforts to avoid missing data will be conducted. The text messages will be sent on a Monday around lunchtime and a reminder will be sent Wednesday if an answer has not been received. The managers of the nursing homes and the supervisors are advised to support text message replies during working hours. Posters and roll ups will be placed at the workplace to remind participants to answer. If answers are still missing, the employees will be called by phone and asked to give their response.

When participants wish to withdraw from the intervention they will be encouraged to make personal contact with the researchers. If they voluntarily give their reason (s) for discontinuing the intervention, these will be registered. A flow diagram describing the dropout rate by clusters will be conducted. Furthermore, analyses to identify possible different baseline characteristics between participants who drop out and participants who continue in the study will be conducted to describe the dropout population and possible risk for confounding.

### Trial status

The trial is ongoing.

## Discussion

Previous studies to improve health literacy have primarily targeted patients or specific deprived groups in health care or community settings. Recently the idea of the workplace as an arena for improving health literacy has developed [23, 29, 33], emphasizing the organizational responsibility in facilitating and supporting that employees obtain basic knowledge and information needed to understand and take action on individual and occupational health concerns [34]. It is predicted that the implementation of health literacy in the workplace context can create value not only for employees, but also for businesses [23, 29, 33]. However the literature about workplace health literacy is still very limited. The development of this intervention is inspired by the apparent potential benefits of improving health literacy in organizations and based on literature about building knowledge and facilitating communication in organizations [57–59].

Previous studies investigating health literacy in workplaces has primarily used a descriptive or exploratory approach, investigating the organizational provision of information about workplace health initiatives and possibilities at the workplace [62] or uncovering/measuring the employee skills to understand and possibilities to act upon health information [63,64]. Thus initiatives to operationalize organizational health literacy need to be developed [34] and evaluated. The existing literature points at the importance of educating employees to be able to access, appraise and apply health information [34, 65] and of organizing the infrastructure and communication in the organization [35]. This study is one of the first to suggest a concrete operationalization of organizational health literacy in a workplace setting. To our knowledge the effort to promote organizational health literacy by empowering both employees and management and structuring organizational processes is new. Through the steps of this intervention the aim is to build a common fundament of knowledge about health and work environment in the organization, and thereby improving the employee and management skills to access information, their competences to understand and appraise these information. In the process of strengthening the individual health literacy the importance of the accessibility and availability of health information and support from the environment has been emphasized [60, 61]. Therefore the aim is to create an environment that supports the use of the obtained knowledge in a continuous communication about and action on health and work environment issues.

It is well-known that maintaining high participation is a difficult process in workplace interventions [16].

This study has a joint focus on both the organization and the individual. Introducing a general course for all employees could potentially generate fellowship among the employees and thereby motivating employees for participation. Furthermore, this approach with multiple steps and facets could embrace some inter-individual differences between employees, in health challenges and needs for health initiatives. In addition we aim to capture the individual needs of each employee through the continuous structured communication between supervisor and each employee about the specific health challenges for the employee, and thereby making it relevant for all employees. Therefore, this intervention offers an innovative alternative to already existing efforts to prevent and reduce pain in considering the several elements on the workplace organization as determinants for pain and consequences of pain in a complex interaction and therefore intervening on the entire organizational knowledge, communication and structures.

Workplace health-promoting initiatives are often time consuming, making it difficult for workplaces with fewer economic resources to launch such initiatives. This initiative is developed to require very limited time from participants and furthermore designed to be normalized in daily routines at the workplaces, making it possible for workplace with limited resources to engage in this intervention.

The intervention is targeting prevention and handling of pain among nurses’ aides, who have high rates of back pain; however, the intervention could potentially be found usable in other job groups with different health challenges.

## Abbreviations

SHS: Social - and health service
NPT: Normalization process theory
CBT: Cognitive behavioral therapy
HLQ: Health literacy questionnaire
